# Irrigation and avifaunal change in coastal Northwest Mexico: has irrigated habit attracted threatened migratory species?

**DOI:** 10.7717/peerj.1187

**Published:** 2015-08-20

**Authors:** Sievert Rohwer, Emily Grason, Adolfo G. Navarro-Sigüenza

**Affiliations:** 1 Burke Museum and Department of Biology, University of Washington, Seattle, WA, USA; 2 Department of Biology, University of Washington, Seattle, WA, USA; 3 Museo de Zoología, Facultad de Ciencias, Universidad Nacional Autónoma de México, México D.F., Mexico

**Keywords:** Thorn forest, Land use change, Avifaunal change, Connectivity, Desert irrigation

## Abstract

Irrigation in desert ecosystems can either reduce or increase species diversity. Groundwater pumping often lowers water tables and reduces natural wetlands, whereas canal irrigation often creates mesic habitat, resulting in great increases in avian diversity from irrigation. Here we compare a dataset of potential natural vegetation to recent datasets from areal and satellite imagery to show that 60% of the land in the coastal plain of southern Sonora and northern Sinaloa lying below 200 m elevation has been converted by irrigation to more mesic habitats. We then use the record of bird specimens in the world’s museums from this same region of Mexico to examine the avian community before and after the development of extensive irrigation. In general these museum records show an increase in the abundance and diversity of breeding birds associated with mesic habitats. Although thorn forest birds have likely decreased in total numbers, most are common enough in the remaining thorn forest that collection records did not indicate their probable decline. Four migrants having most of their breeding ranges in the US or Canada, Yellow-billed Cuckoo, Cliff Swallow, Bell’s Vireo, and Orchard Oriole, apparently have increased dramatically as breeders in irrigated habitats of NW Mexico. Because these species have decreased or even largely disappeared as breeding birds in parts of the US or Canada, further research should assess whether their increases in new mesic habitats of NW Mexico are linked to their declines as breeding birds in Canada and the US For Bell’s Vireo recent specimens from Sinaloa suggest its new breeding population in NW Mexico may be composed partly of the endangered Least Bell’s Vireo.

## Introduction

Biologists generally agree that we are in the midst of an anthropogenic global extinction caused by habitat modification, the introduction of alien species, and global climate change (Wilson, 1992; Steadman, 2006; Jetz, Wilcove & Dodson, 2007; Dirzo et al., 2014). Throughout the Pacific numerous avian extinctions followed the arrival of humans as a consequence of habitat modification, and the introduction of pigs, rats and cats, as well as diseases that island endemics could not resist (Steadman, 1995; Steadman, 2006). Now, on continental landmasses, habitat modification has also become the primary cause of species being listed as threatened or endangered (Wilcove et al., 1998). Even though climate change will be more dramatic in temperate regions, habitat modification and climate change are projected to increase disproportionately as drivers of extinction in tropical species because so many have small ranges, which make them disproportionately susceptible to habitat and climate changes (Jetz, Wilcove & Dodson, 2007).

Here we use historic and recent museum collections to document dramatic changes in the breeding bird community caused by land-use change. During the last half-century massive agricultural irrigation projects have transformed extensive areas of original thorn forest in northern Sinaloa and southern Sonora, Mexico into mesic habitats. This change has the potential to differentially affect species based on habitat preferences. In particular, we were interested to learn whether increased availability of mesic habitat in this region could be providing new breeding habitat for migrant species that are thought to have declined in their historic breeding ranges further north (Wilcove & Wikelski, 2008).

Massive irrigation systems often dramatically transform habitat and bird communities, but these transformations can vary in almost opposite ways. For example, wet meadows and woodlands may disappear when pumping ground water from wells lowers water tables, resulting in substantial declines in the abundance and even the presence of wetland species (Dilts et al., 2012; Yom-Tov, Hatzofe & Geffen, 2012). At the other extreme, irrigation water supplied from large reservoirs via canals usually increases mesic habitats. The forest and shrub habitat, which develop along irrigation lakes, canals and runoff ponds, usually increases diversity by recruiting woodland birds and, sometimes, results in losses of species associated with arid lands (Knopf, 1986; Rahmani & Soni, 1997; Khoury & Al-Shamlih, 2006; Singh, 2009; Yom-Tov, Hatzofe & Geffen, 2012). Missing from these past analyses, however, are considerations of possible linkages between population increases in irrigated regions and population declines in other parts of a species’ range.

Although there are a handful of long-running ecological surveys, few are as old as the earliest collections in natural history museums (Magurran et al., 2010). Thus, the aggregated record of specimens collected during the past two centuries and held by natural history museums constitutes the longest record of population changes against which faunal changes throughout the world can be assessed (Lister Group, 2011). Modern quantitative surveys that include randomized designs and systematic measures of census effort and detectability are limited in time scale; even the venerable Breeding Bird Survey (BBS) of North America was begun only in 1966 (Sauer et al., 2013). In contrast, bird specimen records for Mexico were being accumulated in substantial numbers in the 1880s and, although spotty in geographic and temporal distribution (Navarro et al., 2003), specimens from Mexico have continued to be added to the world’s museums into the 2010s. We use this record of collecting to assess how the avian community of coastal NW Mexico has changed with the development of irrigated agriculture.

This paper first quantifies the extent to which the lowland coastal regions of NW Mexico have been converted to habitats affected by irrigation. This includes not only irrigated fields and their edges, but also pastures that now receive more water as a result of irrigation. Second, we assess how the avifauna changed from an early, pre-irrigation period of collecting (1870s–1970s) to the post-irrigation period of collecting (2000s to 2010s). Finally, we discuss the potential that increasing breeding abundances of some migrants in NW Mexico may be linked to declines in those same species to the north in Canada and the US. In general this study echoes recent admonishments that, without knowing more about the biology of migrant birds in areas south of their normal breeding ranges in the US and Canada, conservation responses to regional population declines may be inappropriate (Wilcove & Wikelski, 2008; Faaborg et al., 2010a; Faaborg et al., 2010b).

### Irrigation in NW Mexico

Federal investments that initiated the construction of small diversion dams and irrigation channels started at several sites of Sonora and Sinaloa in 1934, and lasted until 1970 (Ortega Noriega, 1999). Massive irrigation in coastal NW Mexico escalated after enactment of the Land and Water Reform laws of 1971 redistributed private land in the states of Sinaloa and Sonora to peasants by reducing land-holdings per owner from 100 to 20 hectares in new irrigation districts and distributing the excess land to landless families (World Bank, 1974). With these reforms, a suite of major loans from the World Bank, permitted the construction (1) of large reservoirs on most of the major rivers of coastal Sinaloa and Southern Sonora, (2) of hundreds of kilometers of cement-lined irrigation canals supplying water throughout the coastal plain, and (3) of an extensive network of drains in coastal areas that facilitated agriculture in land near sea level that had previously been too saline to farm.

Before irrigation this region received most of its water as rain that fell during a July through September monsoon (Comrie & Glenn, 1998). Now, when reservoirs are full, this region has access to irrigation water throughout the year and produces crops twice each year in some areas. The demand for water is high and some of the reservoirs in this region are so large (e.g., Huites on the Rio Fuerte) that they do not fill in the annual monsoon. Instead they are filled during typhoons and the water is stored for power and irrigation for several years. When water reserves are low, farmers are advised before the planting season that they must shift to crops demanding less water, with the result that canals are full and irrigation continues even in dry years.

## Methods

### Indexing faunal change

Perhaps the greatest shortcoming of specimen records is that individual records contain no information on the effort invested in obtaining them (Pyke & Ehrlich, 2010). Thus, how much time a collector actually worked in a region is not available for the vast majority of the world’s natural history specimens and we have no measures of the likelihood that a species might be encountered and collected. Although this is a major impediment to the use of specimen records in assessing changes in population densities, it can be partially overcome by using aggregated databases that can be generated through electronic catalogs such as ORNIS (http://www.ornisnet.org/home). We employ an Abundance Index (AI; AIs for multiple values) to convert simple counts of specimens to an index that is corrected by a crude measure of effort (Miki et al., 2000; Rohwer, Navarro & Voelker, 2007; Barry et al., 2009; Rohwer et al., 2012). The measure of effort is the sum of all specimens taken from the same region and time period that would be collected by the methods used to collect the focal species (Barry et al., 2009; Rohwer et al., 2012). The only attempt to evaluate how AIs preform under various sampling senarios was a demonstration that AIs computed as the percentage of adults within a single species in late summer closely paralleled the AIs for those adults computed as percentage of total passerines (Barry et al., 2009).

Formally, AI for a given species is defined as

\begin{eqnarray*} A{I}_{k r}=100\frac{{x}_{k r}}{\sum _{j=1}^{n}{x}_{j r}} \end{eqnarray*}

where *x_kr_* is number of specimens of the *k*th species collected in *r*, the region and time period of interest, and *n* is the number of specimens of all species that would be “expected” to be collected using the same methods in that region and time period of interest. We say “expected” because species included in the numerator and denominator should be about equally as likely to be encountered and collected on a per individual basis. Thus, for example, species that must be collected using song playback should not be compared to species that are readily collected without playback, and nocturnal and diurnal species should not be comparable if they are not encountered and sampled at similar rates. We multiply by 100 to express AI as a percentage.

With the advent of quantitative assessments of morphological variation among populations in the mid 20th century, museums and collectors began attempting to accumulate population samples of most species they were collecting. A consequence of attempting to collect samples suitable for measuring phenotypic variability is that uncommon species that could be sampled in reasonable numbers were likely over-represented relative to their abundance in local communities, whereas common species were likely under-represented. Thus, small changes in AIs should be interpreted cautiously; nonetheless, AIs are surely better measures of faunal change than simple counts of species without correction for effort because the denominator informs us of effort (Rohwer, Navarro & Voelker, 2007; Barry et al., 2009). AIs have previously been shown to track seasonal abundance of migrants, demonstrating that they reliably track regional population changes (Rohwer et al., 2012).

Collecting methods have changed between the two time periods we are comparing. In the early collecting period most specimens were collected with shotgun, while most recent specimens were collected with mist nets. Certainly some birds are easier to collect with nets than with shotguns and vice versa but, as collectors experienced with both collecting methods, our sense is that these biases have minimal effects on total collections. Many individuals of edge and understory species that are over sampled with nets are released so that there is time to prepare good samples of species that are more difficult to collect with nets than with shotgun. Thus, the ultimate goal of acquiring a broad sample of the avian community tends to result in netted and shot birds being sampled in comparable frequencies. Although singing males are easy to collect with shotgun, our experience suggests that biases in species composition are minimal compared to biases in sex ratios generated by these alternate collecting methods. Thus in dense habitats, nets generate far more females than do shotguns, yet the number of individuals sampled per species varies less because collectors are always time-constrained. To minimize biases caused by different collecting methods we have used only cuckoos, hummingbirds, woodpeckers, and passerines to measure effort because most species in these groups are regularly collected with guns or with nets. Although we used a rifle or shotgun for some of our recent collecting, most of these new specimens were netted on joint expeditions by the University of Washington Burke Museum (UWBM) and the Museo de Zoología of the Faculty of Sciences, at the Universidad Nacional Autónoma de México (MZFC). Collecting for this project was conducted under scientific collection permits issued by the Secretaría de Medio Ambiente y Recursos Naturales (SEMARNAT) to the Facultad de Ciencias (B. Hernández-Baños FAUT 0169) of UNAM. All work was conducted in accordance with polices of the University of Washington Institutional Animal Care and Use Committee (protocol 4309-01).

### The Mexican bird atlas

Since the late 1990s, A Navarro and T Peterson have been compiling a database of all the bird specimens in the world’s Natural History museums that have been collected in Mexico (Navarro et al., 2003; Peterson, Navarro-Sigüenza & Benitez-Dias, 1998). There are now more than 370,000 specimens in this database from 71 museums, including the recent collections made by the UWBM and MZFC in coastal Northwest Mexico. All of these records have been georeferenced, making it possible to use them in a GIS framework. From this master database we have extracted a set of specimens appropriate to the questions that led to this paper.

### Study area

In collaboration with MZFC, the UWBM began collecting in coastal NW Mexico to study migrants that move to this region in mid to late summer to undergo their annual molt (Rohwer, Butler & Froehlich, 2005; Pyle et al., 2009). Extensive surveys revealed that most molt migrants were using agricultural fields and thorn forest habitats found below 200 m for molting (S Rohwer, 2014, unpublished data). Molt migrants apparently concentrate in this coastal region of NW Mexico for two reasons. First, the diversity and density of permanent resident species is low because these deciduous forests are mostly leafless during the November through June dry season, resulting in little competition with residents for food during the molt (Rohwer, Butler & Froehlich, 2005). Second, the July through September monsoon turns the deciduous thorn forest green, which generates an abundance of food that is exploited by molt migrants and by other migrants that breed here during the rains but winter elsewhere in Mexico.

We restrict these analyses to areas of southern Sonora and northern Sinaloa, the area of Mexico we collected and surveyed intensively for studies of molt migrants. Fortunately, Chester C. Lamb, a prolific professional collector who worked in Mexico from 1925–1955, lived in Los Mochis, Sinaloa and collected extensively in the same lowland region of coastal NW Mexico that we worked in our recent surveys. These excellent early and recent collections can be used to assess the influence of the recent development of large-scale irrigation on the avifauna of this region.

To make sampling areas comparable for early and recent collections, we eliminated from the full Mexican Atlas (1) specimens from states other than Sonora and Sinaloa, (2) specimens from months other than May–August, (3) specimens taken north of Guaymas in Sonora (27.93°N, 110.89°W) and south of the junction of Route 2 and coastal route 15 in Sinaloa (23.72°N, 106.66°W), (4) specimens collected above 200 m elevation, and (5) a set of specimens that were target-collected for particular research projects and that would have inappropriately inflated either the numerator or the denominator of the AIs. This left us with 2,100 specimens of potential use to the AI analyses. Setting the northern boundary at Guaymas, Sonora means that we include areas around Ciudad Obregón and the Rio Yaki reservoir, which supplies irrigation water to parts of southern Sonora. Most early collectors did not record elevations so the georeferenced data from the Atlas were assigned elevations using a Geographic Information System (ArcGIS 10.2; ESRI). Elevations at each collection locale (m above mean sea level) were extracted from a digital elevation model with 15 m resolution of Sonora and Sinaloa (INEGI, 2013).

Our recent collections included good numbers of birds collected in September, but we omitted September specimens from our analyses for several reasons. First, except for Yellow-billed Cuckoo (*Coccyzus americanus)*, Rufous-winged Sparrow (*Aimophila carpalis*), and Varied Bunting (*Passerina versicolor*), most species collected in September were inappropriate to this analysis of change in the breeding avifauna of this region because most stop breeding in July or August. Second, September marks the beginning of a new influx of species that molted before they migrated south; including these birds would inflate the denominator of the AIs. Third, other local breeders, such as Tropical Kingbirds and Streak-backed Orioles, are much less common in these lowland habitats by September, apparently because most individuals of these species move to other areas in Mexico for their post-breeding molt (Pyle et al., 2009). Finally, early collectors took few September specimens in this region.

### Assessing habitat changes

**Figure 1:**
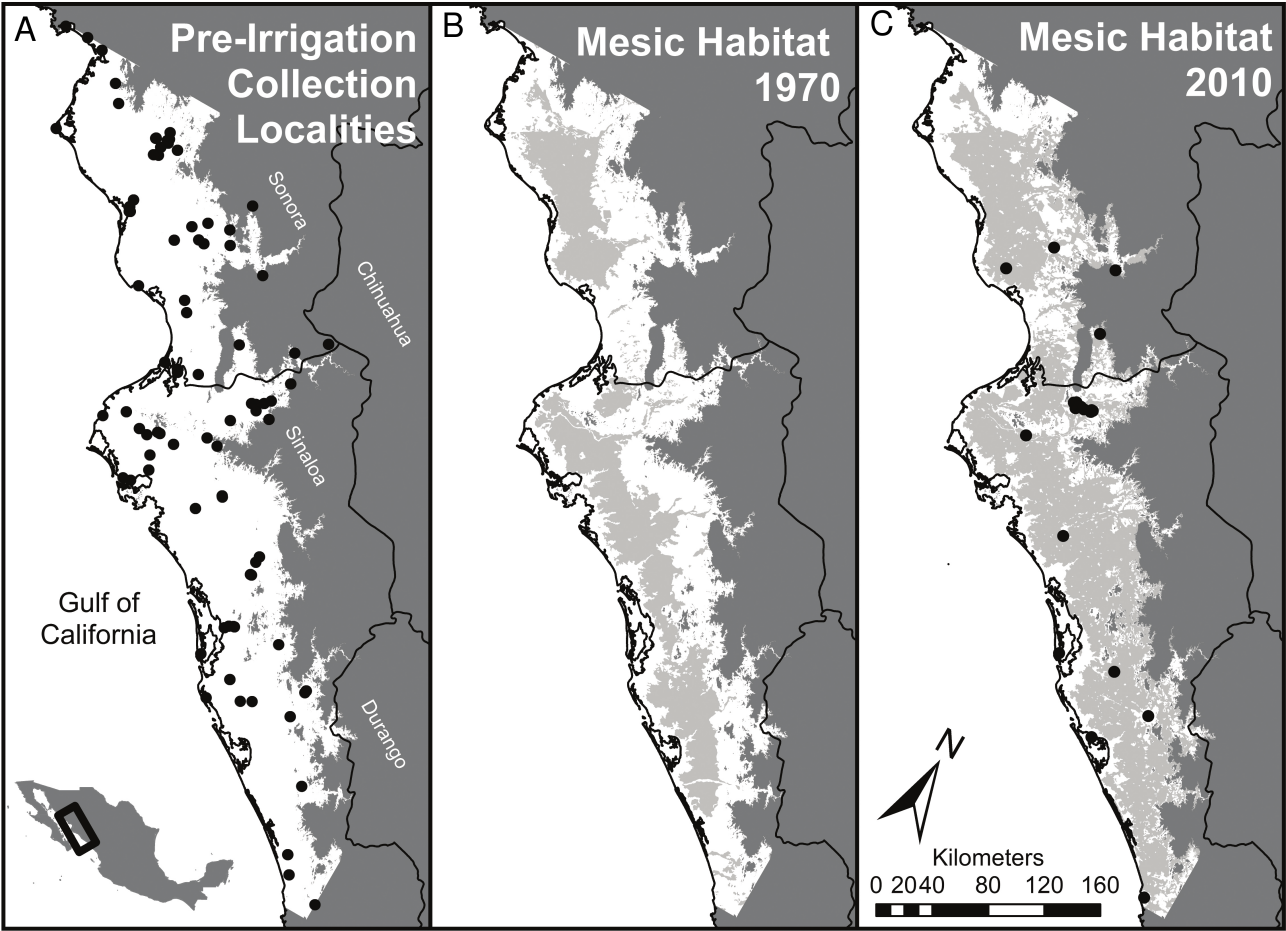
Summary of habitat changes for regions of southern Sonora and northern Sinaloa below 200 m elevation. Early (pre-1970) collecting localities are indicated on (A) and late (1970 to present) collecting localities on (C). (A) represents the study area, a subset of lowland habitat (<200 m above sea level) in southern Sinaloa and northern Sonora. Grey areas of (B) and (C) right panels show the extent of mesic habitat developed by irrigation in this region by 1976 and by 2012, respectively. Coastal wetlands and river corridors were excluded from the mesic habitat designations in the middle and right panels; these areas accounted for 4.4% of the land area below 200 m, most of which was coastal wetlands. This convention means that all of the grey areas in (B) and (C) result from irrigation.

We used a Geographic Information System (ArcGIS 10.2) to quantify the conversion of vegetation types that occurred during the second half of the 20th century as a result of the massive expansion of irrigation. For this purpose, the study area was clipped to include only land below 200m in elevation in Northern Sinaloa and Southern Sonora (bounded by 23.72°–27.93°N, and 106.66°–110.89°W, Fig. 1). Three datasets of land use and vegetation cover, representing different time points, were obtained from the Instituto Nacional de Estadística y Geografía (INEGI, 2015, unpublished data).

#### Pre-irrigation land use

To estimate habitat availability in the study area prior to the installation of major irrigation projects, we analyzed a high-resolution (1:50,000) dataset of potential vegetation types (INEGI, 2013). This dataset was developed from historical imagery and expert analysis, and classifies the land area based on the FAO Land Cover Classification System of vegetation types that would have been present prior to irrigation. We then quantified the amount of this area transformed to mesic habitat by calculating the total area of irrigated land from the following two remotely-sensed datasets, combining land classified as irrigated pasture or irrigated cropland into a single “mesic” category.

#### Land use in 1976

The first land-use dataset (Series I, 1:250,000 scale) was compiled by INEGI during the 1980s, interpreted from areal photography dating, on average, from 1976, and extensively ground-truthed (INEGI, 1980).

#### Land use in 2012

The most recent land-use and vegetation dataset (Series V, 1:250,000 scale) was compiled by INEGI from LandSat data recorded in 2012 (INEGI, 2014).

#### Avifaunal change predictions

To assess changes in species composition that might be attributed to irrigation-related habitat changes in this region we identified two sets of species for comparisons (Table 1). First was a list of 30 species that might have been expected to expand in numbers because they are associated either directly with mesic habitats or because they prefer habitats with larger trees for breeding. Formerly, large trees were found only along rivers in coastal west Mexico, but they are now found around numerous seeps or lakes formed where irrigation canals cross what, previously, had been dry arroyos. Irrigation canals are built along elevation contours that are cut by arroyos. Where modern concrete-lined irrigation canals cross small arroyos a concrete siphon is typically built under the arroyo, resulting in no loss of water, but where these canals cross wide arroyos the lower side of the arroyo is simply dammed with earth, creating a lake that may be quite large. Large trees and dense brush often grow around these lakes and, particularly, in the seeps below their earth dams. Several or the 30 mesic species noted in Table 2 were likely under represented in our recent collecting because they are easier to collect with shotguns than nets (e.g., some tyrannids, swallows, grassquit). We retained these species because some warrant further consideration and because the variability in AI ratios around ±1 (see below) helps inform us about the importance of species showing larger changes in relative abundance over time.

**Table 1:** Thorn forest and mesic species used in the AI analyses with breeding status indicated by month. Breeding was assessed from field observations of behavior and nests, and from the condition of collected specimens. “No” signifies species that were not breeding and blanks signify species for which we had too few observations to assess active nesting.

	May	Jun	Jul	Aug
Thorn forest species				
*Aimophila carpalis*	No	x	x	x
*Auriparus flaviceps*	x	x	x	
*Campylorhynchus brunneicapillus*	x	x	x	x
*Colaptes auratus/chrysoides*	x	x	x	
*Geococcyx californianus*	x	x	x	x
*Passerina versicolor*	No	x	x	x
*Pheucticus chrysopeplus*	x	x	x	x
*Pipilo fuscus*	No	x	x	
*Polioptila melanura*	x	x	x	x
**Mesic species**				
*Agelaius phoeniceus*	x	x	x	No
*Cacicus melanicterus*	x	x	x	No
*Calocitta colliei*	No	x	x	x
*Coccyzus americanus*	No	x	x	x
*Corvus sinaloae*	x	x	x	x
*Crotophaga sulcirostris*	No	x	x	x
*Geothlypis trichas*	x	x	x	x
*Hirundo pyrrhonota*	x	x	x	No
*Icteria virens*	x	x	x	x
*Icterus pustulatus*	x	x	x	No
*Icterus spurius*	x	x	x	x
*Melospiza melodia*	x	x	x	No
*Mimus polyglottos*	x	x	x	No
*Molothrus aeneus*	x	x	x	
*Molothrus ater*	x	x	x	x
*Myiarchus tyrannulus*	x	x	x	No
*Myiodynastes luteiventris*	x	x	x	No
*Myiozetetes similis*	x	x	x	
*Pachyramphus aglaiae*	No	x	x	Mostly no
*Pitangus sulphuratus*	x	x	x	
*Quiscalus mexicanus*	x	x	x	
*Saltator coerulescens*	x	x	x	
*Sayornis nigricans*	x	x	x	
*Stelgidopteryx serripennis*	x	x	x	No
*Thryothorus felix*	x	x	x	x
*Thryothorus sinaloa*	x	x	x	x
*Tyrannus melancholicus*	x	x	x	Mostly no
*Oreothlypisluciae*	x	x	x	No
*Vireo bellii*	x	x	x	No
*Volatinia jacarina*	?	x	x	x

**Table 2:** Numbers of individuals collected in pre and post irrigation periods for focal species and their AI values. The final numeric column presents AI ratios, with increases indicated by positive numbers and decreases indicated by negative numbers (see text for details).

	Early collecting						Recent collecting							
Species	May	Jun	Jul	Aug	Total	AI	May	Jun	Jul	Aug	Total	AI	AI change	Notes on potential collecting bias
**Decrease predicted**														
*Aimophila carpalis*	9	9	1		19	1.57		3	10	7	20	2.25	1.44	
*Auriparus flaviceps*	8	6	1		15	1.24		2	10	2	14	1.58	1.28	
*Campylorhynchus brunneicapillus*	2	4	1		7	0.58	1	1	2	7	10	1.13	1.95	
*Colaptes chrysoides*	5	8	2	1	16	1.32		1	2		3	0.34	−3.90	
*Geococcyx californianus*	2	5		1	8	0.66		1	1		2	0.23	−2.87	
*Passerina versicolor*	7	10	1		18	1.48	3	13	16	2	31	3.49	2.36	Breeds in thorn scrub but abundant forager in weedy edges.
*Pheucticus chrysopeplus*		12			12	0.99			1		1	0.11	−8.77	
*Pipilo fuscus*	3	4			7	0.58			2		2	0.23	−2.56	
*Polioptila melanura/nigriceps*	6	7	0	4	17	1.40			8	5	13	1.47	1.05	
Totals/Mean AI values					119	1.09					96	1.20	1.02	
**Increase predicted**														
*Agelaius phoeniceus*				1	1	0.08	2	2			2	0.23	2.74	Abundant breeder in cattails where we seldom collected
*Cacicus melanicterus*		27			27	2.23		4	1		5	0.56	−3.95	Under sampled with nets. The 27 early specimens were taken in June 2004 at a single locality.
*Calocitta colliei*	1	6			7	0.58			2	2	4	0.45	−1.28	Under sampled with nets.
*Coccyzus americanus*		3			3	0.25			12	1	13	1.47	5.93	Settles in late June, breeds Jul–Aug. The three early specimens, taken 9, 14, 22 Jun, are likely migrants.
*Corvus sinaloae*	8	10	1	4	23	1.90		1	5	7	13	1.47	−1.29	Under sampled with nets.
*Crotophaga sulcirostris*	1	7	2	8	18	1.48			8	3	11	1.24	−1.20	
*Geothlypis trichas*	6	7			13	1.07		1	1	1	3	0.34	−3.17	Common near coast where we seldom collected.
*Hirundo pyrrhonota*		3		1	4	0.33		6	3	2	11	1.24	3.76	Large colonies under most bridges.
*Icteria virens*	5	10		4	19	1.57			14	12	26	2.93	1.87	Common in brushy edges.
*Icterus pustulatus*		18	1		19	1.57		6	6	8	20	2.25	1.44	
*Icterus spurius*		4		1	5	0.41		1	26	4	31	3.49	8.48	Now an abundant breeder.
*Melospiza melodia*		2			2	0.16	1	2	4		6	0.68	4.10	Common along coastal canals.
*Mimus polyglottos*				1	1	0.08	3	4		5	9	1.01	12.31	Now an abundant breeder in ecotones.
*Molothrus aeneus*	9	8	1	2	20	1.65		5	4		9	1.01	−1.62	
*Molothrus ater*	3	4		1	8	0.66		1	3	9	13	1.47	2.22	August increase could be birds moving in to exploit late breeding hosts.
*Myiarchus tyrannulus*	13	9			22	1.81		4	3	5	12	1.35	−1.34	Under sampled with nets.
*Myiodynastes luteiventris*		5			5	0.41		1			1	0.11	−3.66	Under sampled with nets.
*Myiozetetes similis*	7	8		4	19	1.57		2	4		6	0.68	−2.32	Under sampled with nets.
*Pachyramphus aglaiae*	8	12		6	26	2.14			4		4	0.45	−4.75	Under sampled with nets.
*Pitangus sulphuratus*	4	6	1	3	14	1.15		1	5		6	0.68	−1.71	Under sampled with nets.
*Quiscalus mexicanus*	4	7	3		14	1.15	7	8	16	7	31	3.49	3.03	Many nesting colonies near canals close to coast.
*Saltator coerulescens*	4	4		5	13	1.07		4	6		10	1.13	1.05	
*Sayornis nigricans*		2			2	0.16		3	2		5	0.56	3.42	Under sampled with nets. Common along canals.
*Stelgidopteryx serripennis*		3			3	0.25			10	8	18	2.03	8.21	Common along canals.
*Thryothorus felix*	2	7	1	1	11	0.91		3	11	8	22	2.48	2.74	
*Thryothorus sinaloa*	1	7			8	0.66			2		2	0.23	−2.92	
*Tyrannus melancholicus*	11	19	2	5	37	3.05		4	15	10	29	3.27	1.07	Under sampled with nets.
*Oreothlypis luciae*		4		1	5	0.41		3	21	3	27	3.04	7.38	Now an abundant breeder in mesquite forest.
*Vireo bellii*	2			1	3	0.25		1	9	13	23	2.59	10.48	Now an abundant breeder in ecotones.
*Volatinia jacarina*		3	1		4	0.33			3	5	8	0.90	2.74	Now common in weedy fields. Flies through nets, so under sampled in recent collecting.
Totals/Mean AI values					356	0.98					380	1.43	2.97	

Our second list was nine species that mostly breed in the deciduous thorn forest that previously dominated this region of Mexico (Table 1). Because of the massive decline in the extent of thorn forest, these species might be expected to have declined in total numbers throughout the region, but collecting bias tends to nullify this expectation. Collectors generally try to obtain at least small samples of all species found breeding in a region and we certainly paid attention to thorn forest specialists because they were under represented in the UWBM collections. Although thorn forest specialists have likely declined in total numbers in coastal NW Mexico, enough thorn forest habitat persists in areas without irrigation that samples of most thorn forest breeders are easily obtained. For this reason, declines in relative numbers of thorn forest species could only be expected in exceptional cases.

Our collected specimens and field observations showed that all species in both categories were breeding in June and July; some species had not yet begun breeding in May, and others were no longer initiating nests in August, although adults and fledged young were present in August (Table 1). Just three of the focal species were recorded breeding in September, Greater Roadrunner (*Geococcyx californianus*), Yellow-billed Cuckoo, and Rufous-winged Sparrow. When our observations were insufficient to determine breeding status during certain months those cells in Table 1 were left blank.

### Plotting AI ratios

When one component of a community is expected to increase between two time periods (our mesic species) and another is expected to remain the same or to decrease (our thorn forest species) potential changes in their AIs need to be presented in a way that scales increases and decreases in AI values equally. We have done this by computing ratios of the AI values for the pre- and post-irrigation collecting periods. When AI values for a species increased between early and late collecting periods, they are expressed as positive ratios, AI_*l*_/AI_*e*_, where the subscripts *l* and *e* refer to late and early collecting periods, respectively. When AI values for a species decreased between the early and late period they are plotted as negative ratios, AI_*e*_/AI_*l*_. Interchanging the numerator and denominator in these ratios avoids a ratio that is bounded by 0, thus scaling relative increases and decreases equivalently.

When histograms of AI ratios are used to index faunal change, species clustering near ±1 assess volatility in AI ratios that, at least in part, are driven by sampling error. This focuses attention on large changes in AI ratios, which represent species whose numbers are most likely to have changed through time. Assuming collectors attempt to assemble samples of as many species as possible, species present in sufficient numbers to be reasonably represented in two collecting periods should show little change in their AI ratios. In contrast, outliers should be species that either were so uncommon that reasonable samples could not be obtained, or that were so common that they were over-sampled when less common species were not being caught. Thus AI ratios falling beyond the peak in the distribution centered on ±1 should most reliably indicate population changes.

## Results

### Land use change

The extensive development of irrigation in coastal NW Mexico has dramatically increased mesic habitat. Irrigation was taking place in this region on a local scale prior to the 1970s, but the scale of that irrigation was trivial compared to the transformation of this region following the agrarian reform laws of 1971. Irrigated agriculture on an industrial scale now accounts for the vast majority of land use on the coastal plain of northwest Mexico.

The projected natural vegetation for the region (outlined in Fig. 1) was primarily spiny thorn forest (38%) and dry scrublands (35%) as well as deciduous forest (21%). Mesic habitat was found only in areas with riparian vegetation, and comprised less than 5% of the land cover prior to irrigation. By the time of the first land-use assessment for Mexico in 1976 approximately 34% (1,293,231 hectares) of the low elevation areas of NW Mexico were categorized as mesic habitats (Fig. 1B). This was mostly due to small diversion dams and local irrigation projects and that were constructed before loans from the World Bank helped build much larger reservoirs and cement lined canals. Now, 60% of the land area (2,293,766 hectares) in our study region of coastal NW Mexico is currently covered by mesic habitat (Fig. 1C). Areas that remain unaffected by irrigation are largely hills and small mountains with slopes unsuitable for agriculture and desert habitats on the coastal plain that cannot, at present, be supplied with water.

### Faunal change

Most collecting that occurred in coastal northwest Mexico prior to the development of modern irrigation occurred before the 1970s (Table 3). Then, there was a substantial to complete hiatus in collecting from the 1970s through the 1990s, during which time many major irrigation dams and canals were built. Collecting resumed with the joint UWBM/MZFC expeditions to this region conducted from 2003–2011. Of the 2100 specimens in the analysis, 1,213 were collected during the early period (Pre 1970s), predating the majority of land-use change, and 887 have been added since (Table 3).

**Table 3:** Number of specimens taken per decade from coastal regions of southern Sonora and northern Sinaloa below 200 m from May through August. Most of the specimens from the 1930s were collected by Chester C. Lamb, who lived in Los Mochis and collected extensively in areas worked by the UWBM from 2003–2011. Preirrigation collecting occurred earlier in the season than the recent UWBM collecting.

Decades	May	Jun	Jul	Aug	Totals
1870s	0	0	1	0	1
1880s	16	6	0	1	23
1890s	1	1	2	0	4
19002	2	77	1	0	80
1910s	1	0	0	0	1
1920s	7	40	1	0	48
1930s	233	273	173	89	768
1940s	32	0	0	0	32
1950s	0	66	6	0	72
1960s	5	106	26	18	155
1970s	26	0	2	1	29
**Early totals**	**323**	**569**	**212**	**109**	**1213**
1980s	0	1	0	0	1
1990s	0	0	0	0	0
2000s	0	0	433	276	709
2010s	25	152	0	0	177
**Recent totals**	**25**	**153**	**433**	**276**	**887**
					
**Grand totals**	**348**	**722**	**645**	**385**	**2,100**

We found no change in the rate at which the group of nine thorn forest species were collected before and after irrigation (Table 4; Fisher’s exact *p* = 0.28). AI values normalized by the number of species were 1.09 and 1.25, for the early and recent periods respectively (Table 4). In contrast, there was a significant increase in the normalized AI values for the group of 30 mesic species from 0.98 in the early period to 1.48 in the post-irrigation period (Table 4; Fisher’s exact *p* < 0.0001).

**Table 4:** Changes in pooled AI values split by thorn forest and mesic species. The final numeric column presents AI ratios, with increases indicated by positive numbers and decreases indicated by negative numbers (see text for details).

	Number of individuals	All specimens	Normalized AI values (per species AI)	Chi-square (Yates correction)
**Thorn forest species (9 species)**				
Early collecting	119	1,213	1.09	*X*^2^ = 0.82
Recent collecting	100	887	1.25	*p* = 0.54
**Mesic Species (30 species)**				
Early collecting	356	1,213	0.98	*X*^2^ = 22.95
Recent collecting	380	887	1.48	*p* < 0.0001

**Figure 2:**
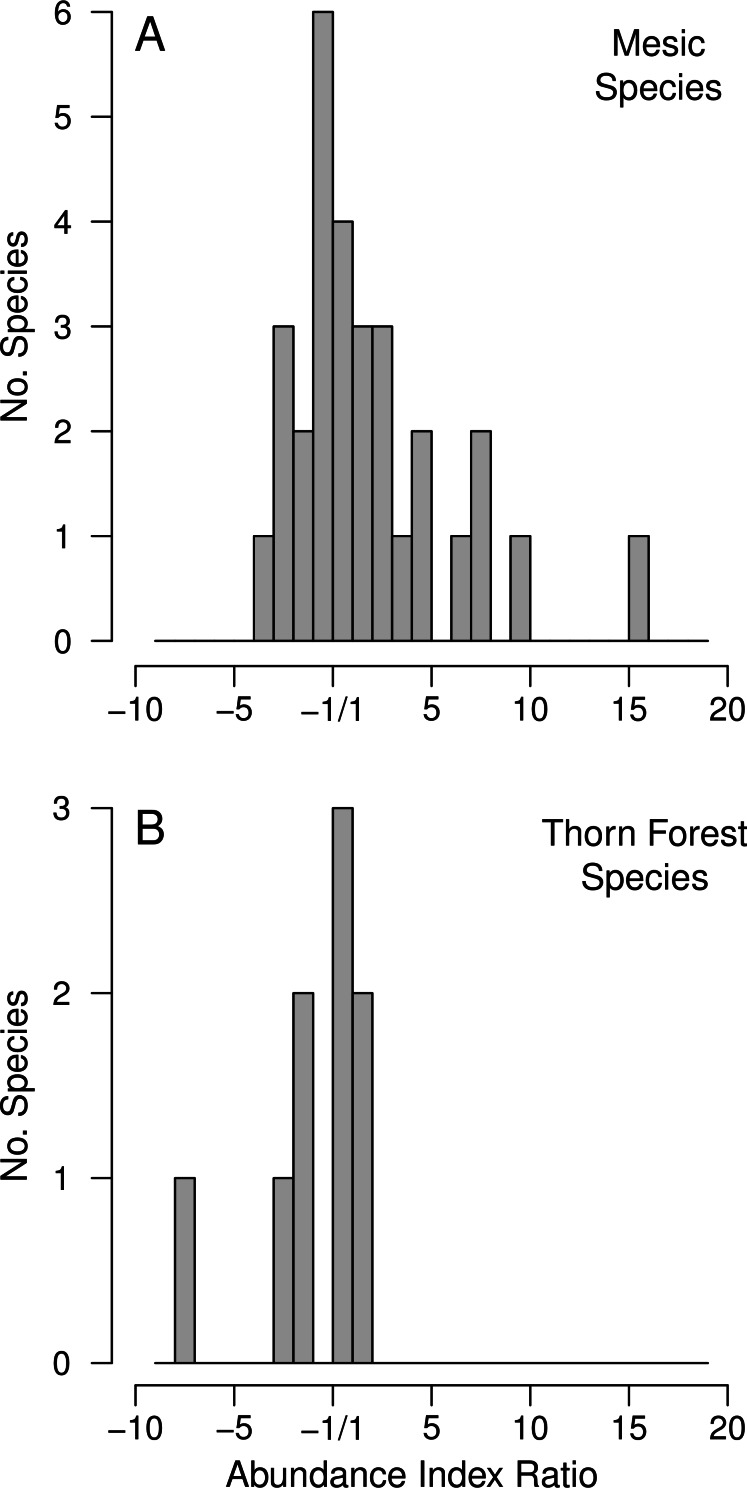
Histograms of the distribution of the AI ratios for the thorn forest taxa (9 species) and for the species associated with mesic habitats or large trees (30 species). Positive values represent species that have increased in relative abundance from the pre- to post-irrigation period and negative values represent species that have decreased. Note that these ratios cannot fall between ±1. Large changes are most likely to reliably indicate actual changes in relative population sizes, but AI values closer to ±1 may largely reflect random volatility in the index.

Examining changes in the AI ratios between the early and late periods of collecting (Table 2) provides further insight into the volatility of these ratios. There is a great deal more variance in the AI ratios for mesic than for thorn forest species (Fig. 2). More importantly, there is a strong right skew in the ratios for mesic species because some mesic species were seldom collected in the early period, but frequently collected in the post-irrigation period. Although collectors generally attempt to evenly sample species in a community, this skewed distribution suggests that moderate increases for a number of mesic species were, nonetheless, reflected in museum collections.

For the nine thorn forest taxa AI ratios are, as expected, little skewed (Fig. 2). Yellow Grosbeak (*Pheucticus chrysopeplus*) is the most negative point in this distribution, and likely has declined in total numbers at low elevations in coastal NW Mexico (Table 2; Fig. 2). Yellow Grosbeaks are never abundant in collections, and they were uncommon enough that we had little sense of their habitat requirements. We certainly over-sampled Yellow Grosbeaks, compared to their actual abundance. It was the only thorn forest species for which we collected every individual captured but densities were too low for us to capture reasonable samples.

### Individual species

A few species, including two that we did not treat in the AI analyses, merit further descriptive comment and interpretation. All may have increased as a consequence of irrigation.

#### Athene cunicularia

No Burrowing Owls were collected in our work in coastal NW Mexico. However, we observed several nesting pairs about 12 km S and 2 km W of El Carrizo, Sinaloa. Prior to extensive clearing of thorn forest and the development of irrigated agriculture this species wintered in coastal NW Mexico but was not reported to breed there (Enriquez-Rocha, 1997). The Mexican Atlas includes no May–September specimens from the pre-irrigation period, but numerous wintering specimens; now they breed abundantly in burrows they excavate in the banks of coastal drains. Macias-Duarte (2011) quantified breeding densities of Burrowing Owls in coastal Sinaloa and Sonora and found densities among the highest ever recorded. Now it is presumably a common breeder because of the ease with which burrows can be excavated in the banks of coastal drains.

#### Ortalis wagleri

We excluded the Rufous-bellied Chachalaca from our AI analyses because it cannot be collected with nets, but we did obtained reasonable samples of this bird with a .22 we borrowed for collecting. Prior to 1970 the atlas database contains seven May–August specimen records from Coastal NW Mexico, but there are 22 records for the same months since 2007. Pairs are now found everywhere along canals and in arroyos where flowing water or seeps below dams have stimulated the development of larger trees and dense brush. We did not find it in thorn forest away from large trees or dense brush. There can be little doubt that this resident chachalaca has increased enormously in response to the increase in dense vegetation associated with irrigation.

#### Coccyzus americanus

Just three Yellow-billed Cuckoo specimens were taken in the early period of collecting, and all were taken in early to mid June before this species begins to breed in the lowland habitats we worked (Rohwer & Wood, 2013). All of the early to mid June specimens we collected were migrants and territorial birds were not found until late June (Rohwer & Wood, 2013). Although Yellow-billed Cuckoos bred at higher elevations in NW Mexico before irrigation (Short, 1974), they surely have increased dramatically as breeding birds in the coastal lowlands in response to irrigation.

#### Passerina versicolor

The Varied Bunting is the only thorn forest species to show a moderate increase in the rate at which it was collected in recent years. We categorized this species as a thorn forest bird because it can be found breeding in thorn forest far from irrigated fields. However, it also breeds in brushy ecotones, and it commonly forages in sorghum fields and in the weedy edges of other irrigated fields where it was frequently netted. We released many individuals, suggesting that this bunting was under sampled relative to its actual abundance, even though students are eager to prepare stunning adult males and age-related plumage variation makes this species particularly interesting. This species has likely increased in total numbers because sorghum fields and weedy edges offer so much food.

#### Petrochelidon pyrrhonota

Cliff Swallows were little collected in the pre irrigation era and were probably mostly restricted to towns where they would have nested on buildings. Throughout irrigated areas of the coastal region we found nesting colonies under the bridges crossing most of the coastal drains, under many river bridges, and on structures near water that create foraging and mud gathering habitat.

#### Icterus spurius

Early (Friedmann, Griscom & Moore, 1950; Miller et al., 1959) and more recent faunal works (Howell & Webb, 1995) were unclear about the status of Orchard Orioles as a breeding bird in coastal NW Mexico. The Atlas contains only 5 pre-1970s specimens, 4 from June and 1 from August, but the age and breeding status of these specimens has not been addressed. The June specimens could be breeding birds, but the peak of nesting now occurs in July (Rohwer et al., 2012) when no specimens were taken in the pre-irrigation period. Even if the early specimens were all breeders this species has surely increased dramatically following the widespread development of irrigation. Breeding individuals and small colonies are now common, particularly where appropriate nest trees are found adjacent to irrigated alfalfa fields (Rohwer & Wood, 2013).

#### Melospiza melodia

Although two June Song Sparrows were taken in the early collecting period, this bird is emphatically reported not to breed in this region of Mexico by Howell & Webb (1995). However, it is now a common breeder along the brushy edges of coastal canals and drains, and pairs can even be found at edges of mangrove stands that have developed enough soil and tidal litter to support patches of low “forbes” for foraging and nesting cover.

#### Sayornis nigricans

We seldom collected Black Phoebes because they usually perched and foraged over canals where it was difficult to set nets. Although never abundant, they have surely increased dramatically; they nested under bridges over drains and canals throughout the region.

#### Stelgidopteryx serripennis

We are uncertain that Northern Rough-winged Swallows have increased as a breeder in this region because the species is also a molt migrant from the north, with the earliest molting birds arriving in July (Rohwer, Hobson & Yang, 2011). Estimating the frequency of molt migrants from the north among the hundreds of July and August birds often seen around the canals and lakes would require more isotope work on birds that are actively molting.

#### Oreothlypis luciae

Lucy’s Warbler had not previously been recorded as a breeder in Mexico except near the US border (Howell & Webb, 1995; Johnson, Yard & Brown, 2012). However, an abundance of singing and territorial males that were mated in May and June show that Lucy’s Warblers are now common breeders in stands of larger mesquite trees that have grown up near water sources. Because we did little collecting in May and June and because we seldom netted in habitats where Lucy’s Warblers breed, the early years of our recent collecting seriously under-represent breeding abundance of Lucy’s Warblers in Sinaloa. Most of our specimens were taken in July and August in a grassy mesquite flat that was part of an irrigated pasture and attractive to molt migrants. We assumed these Lucy’s Warblers were also molt migrants (Rohwer, Navarro & Voelker, 2007), but the newly documented status of this bird as a common breeder in northern Sinaloa, suggests these molting individuals could have been local breeders and locally produced young. Nonetheless, individuals from the north still seem to move here to molt because we observed apparent migrants moving south through the desert in Sonora on 9 July 2006, before they begin the molt (Rohwer, Navarro & Voelker, 2007).

#### Vireo bellii

For the principal breeding months of June and July, there are no Bell’s vireo specimens from coastal NW Mexico, but there are two records from May that could have been breeders or migrants. Now this species breeds abundantly in brushy edges around most irrigated fields, and there is little doubt that it has increased enormously as a breeding bird in this region. Our records for this species may somewhat overestimate its change in abundance because we collected a number of puzzling birds that were singing in late summer, most of which turned out to be males hatched that season; some had partially developed testes.

For the Bell’s Vireos now breeding in coastal Sinaloa, source populations could have been either the endangered *V. b. pusillus*, which now breeds only in southern California and northern-most Baja, or *V. b. arizonae*, which breeds in Arizona and Nevada. These races are difficult to distinguish in fresh fall plumage. Thus, SAR compared 25 new specimens from Sinaloa with reference material of *pusillus* collected in California and the Baja peninsula (*n* = 25) and *arizonae* collected Arizona (*n* = 17). Breeding season *pusillus* from California largely lack the yellowish wash on their flanks that characterize most *arizonae*, but *pusillus* in fresh plumage from coastal California (MVZ 104087, 4057, 3353, 37144) and from Baja Sur (MVZ 44325 and 55750; UWBM 81277) were as yellowish on their flanks as many *arizonae*. For recently collected Sinaloa specimens, those that matched or were more greenish yellow on their flanks than these seven reference specimens were considered *arizonae* and paler specimens were considered *pusillus*. By this criterion, 18 of the new Sinaloa specimens were considered arizonae and 7 were considered *pusillus*. We should emphasize that these difference are subtle and, as is often the case with continental races, many individuals could not be assigned to race with confidence.

## Discussion

The changes in the breeding avifauna that we have documented emphasize two important points. First, following the onset of irrigation, mesic habitats have increased from less than 5% of the land area to more than 60%, and this change has substantially changed the composition of the avian community of coastal NW Mexico. These changes are similar, though perhaps less dramatic to those seen in many other desert regions of the world where raised water tables and consistent flow of water through irrigation canals often transforms arid lands to mosaics of wetlands and mesophilic vegetation, resulting in increases in birds associated with mesic habitats (Knopf, 1986; Rahmani & Soni, 1997; Khoury & Al-Shamlih, 2006; Singh, 2009; Yom-Tov, Hatzofe & Geffen, 2012). Second, generating AIs by tallying all specimens from a region and time period that could reasonably be expected to have been collected by the same methods used to collect the focal species corrects, at least partially, for differences in effort; thus, AIs more reliably track changes in densities than do counts of specimens. Incorporating data on age, fat, and the breeding status of specimens into electronic data repositories would greatly increase the utility of specimens to studies of population biology.

Early avian collecting from 1880–1970 in coastal NW Mexico predated the development of intensive irrigation, while recent collecting, mostly from 2002–2012, occurred after massive irrigation was well developed. Thus the AIs we computed for early and recent collecting in NW Mexico assess avifaunal change across with this dramatic increase in mesic habitats. Although collectors likely over-collect rare species and under-collect common species, time constraints on collectors together with the demand to generate good numbers of specimens mean that the relative abundance of species available for sampling will influence the relative numbers of individuals collected, resulting in AIs at least crudely indexing changes in abundance.

Some species that we found to have increased were sampled prior to the initiation of massive irrigation programs in the 1970s, but they were taken in small relative numbers; moreover, collection dates suggest that some pre-irrigation specimens were migrants. Only a single Red-winged Blackbird (*Agelaius phoeniceus*) and a single Northern Mockingbird (*Mimus polyglottos*) were collected in this region of Mexico prior to 1970, and both were August specimens that were likely post-breeding immigrants (Table 2). Now Red-winged blackbirds commonly nest in cattails that grow around lakes formed by canals and along coastal drains that reclaim saline lands for agriculture. Similarly, mockingbirds are now common breeders around the brushy edges of irrigated fields and were frequently caught in our nets (Table 2). The increases suggested for these species by our AI ratios surely underestimate their increase as breeding birds; we shot no redwings and they were seldom netted, and we released many netted mockingbirds. In contrast, there are three early specimens of Yellow-billed Cuckoos from this region, but all were collected in early June when migrant cuckoos are present but before they have are settled on territories in this region of Mexico. For consistency these three early specimens were included in the early AI but, if there were migrants, as recent collections suggest, then the AI ratio suggests less increase in cuckoos than likely has occurred.

By comparing current land use with potential natural vegetation we estimated that 60% of the low thorn forest of NW Mexico was converted to mesic habitat. This landscape change has resulted in the apparent addition of several new breeding birds to this region of Mexico (Burrowing Owl, Yellow-billed Cuckoo, Lucy’s Warbler, Orchard Oriole), and to dramatic increases in other species that were either of uncertain status or uncommon as breeders prior to irrigation (Cliff Swallow, Bell’s Vireo, Northern Mockingbird, Red-winged Blackbird). Thus irrigation has increased the diversity of the breeding avifauna of this region. At the same time, about 40% of the land area in this region is on slopes that cannot be irrigated, and this remaining dry habitat continues to support most of the arid-land species that predominated in this region prior to massive irrigation. Indeed, Yellow Grosbeak was the only thorn-forest specialist for which there were few enough recent specimens to suggest a decline in its numbers. Although other thorn-forest species have surely declined in total numbers from loss of habitat, the remaining thorn forest is extensive enough that they are easily collected.

### Conservation implications of expanded breeding numbers in Mexico

Could population changes in migrants breeding in Mexico and the US linked? When a species is winter limited (Fretwell, 1972; Terborg, 1989), the development of either higher quality habitat in NW Mexico, or of habitat similar in quality to historical breeding areas to the north that reduces migration distance, may result in a decline in numbers to the north. In contrast, if populations are regulated by density dependent breeding success, colonization of new breeding habitat in one region should not reduce breeding numbers elsewhere, as long as the winter range can accommodate summer production. Asymmetries in the land areas of breeding and wintering ranges for Neotropical migrants suggest winter limitation could be the rule; their winter range is often only a third to a fifth the size of their summer range and they arrive there with numbers swollen by summer production (Terborg, 1989).

We seldom know whether populations are primarily winter or breeding season limited; yet, most studies of threatened and endangered birds focus on losses of breeding habitat and cowbird parasitism (e.g., Goldwasser, Gaines & Wilbur, 1980; Gaines & Laymon, 1984; Kus, 1998), which implicitly assumes breeding season regulation. However, migratory shortstopping and winter population regulation can easily lead to population declines in northern breeding areas that are driven, not by losses of northern breeding habitat, but by the development of better habitat to the south. Such population shifts need not be driven solely by migratory shortstopping. Certainly, pioneering breeders in the south must be shortstoppers but, as soon as recruitment is higher in the south than in the north, increases in numbers in the south will be matched by declines in the north, if populations are winter limited.

Flexibility in the settlement patterns of Neotropical migrant passerines has been documented to occur over vast geographic scales, apparently through domino effects of territorial behavior. In Black-throated Blue Warblers (*Setophaga caerulescens*) adults are concentrated in the southern Appalachian Mountains where their density and productivity is high, whereas yearlings predominate to the north over large areas where densities are low (Graves, 1997). Yearling American Redstarts (*Setophaga ruticilla*) that depart early from their Jamaican wintering grounds settle south of their birth place in North America, but yearlings that depart late settle north of their birth place, presumably because southern habitats have filled with earlier migrants. Moreover, these distances are related to their relative departure dates (Studds, Kyser & Marra, 2008). Similarly, as measured by age ratios, the habitat distribution of Hermit and Townsend’s Warblers (*Setophaga occidentalis* and *S. townsendi*, respectively) in the Cascade Mountains of Washington and Oregon are strongly affected by elevation gradients. At low elevations adult Townsend’s Warblers show strong breeding site fidelity (Pearson, 2000), and adults at low elevations that fail to return in spring are replaced, not by yearlings, but by unmarked adults. Yearling males settle at high elevations for their first breeding season (Rohwer, 2004) but apparently moved to more suitable low-elevation sites after breeding their first season at high elevations (Pearson, 2000; Rohwer, 2004).

Most studies of the decline of migrants breeding in the American Southwest have focused almost exclusively on habitat loss in that region, probably because most of the money available for these studies must be spent in the US. This is distressing: these migrants spend the majority of the annual cycle south of the US and their population biology is little studied outside of the breeding season. If there is winter population regulation and if breeding populations in the US and coastal NW Mexico are linked by migratory shortstopping, then restored habitat in the US would need to exceed the quality of the new breeding habitat in NW Mexico sufficiently to repay the cost of a longer, trans-desert migration. The huge increase in mesic habitat, from less than 5% to 60% of the land area in coastal NW Mexico has resulted in large new breeding populations of some migrants that could be linked to declines in the US.

Eight long-distance migrants included in our assessments of changes in breeding abundance in the irrigated regions of coastal NW Mexico are reasonable candidates for such linkages (Table 5). Two of these eight species, Northern Rough-winged Swallow and Lucy’s Warbler, are molt migrants to this region (Rohwer, Navarro & Voelker, 2007; Rohwer, Hobson & Yang, 2011). For this reason the increases we estimated for these species may be high, so we do not further consider these species. Two others, Yellow-breasted Chat (*Icteria virens*) and Common Yellowthroat (*Geothlypis trichas*), appear to have changed little in abundance in coastal NW Mexico and neither shows BBS (Sauer et al., 2013) declines in the west (Table 5). Chats have likely expanded with the increase in brushy edges, but they are well represented in collections prior to the 1970s; our recent collections seldom included breeding yellowthroats, which were common only along the coast (Table 2). In contrast, Yellow-billed Cuckoo, Cliff Swallow, Bell’s Vireo and Orchard Oriole, have likely increased dramatically as breeders in the irrigated regions of NW Mexico. All are migrants with breeding ranges mostly in the US and Canada and all have declined to the north, suggesting their population changes could be linked.

**Table 5:** Migrant species breeding in NW Mexico that have most of their breeding range in the US and Canada. Positive AI ratios signify an increase in the relative frequency of specimens in collections from coastal Sinaloa and Sonora after extensive development of irrigation; Breeding Bird Survey trends are from the 1966 to 2011. For the first four species we suggest that population increases in Mexico may be linked to population declines in the US and Canada.

Species	AI ratio	Population trends to the north	Notes for irrigated regions of NW Mexico
*Coccyzus americanus*	+5.93	Mostly lost in the west; BBS shows significant declines in most of its eastern range.	Common breeder; seldom netted, but some collected with gun.
*Petrochelidon pyrrhonota*	+3.76	BBS: significant declines in the west; sharply declining in Canada.	Seriously under-sampled in our recent collecting in NW Mexico.
*Vireo bellii*	+10.48	*Vireo belli pusillus* is mostly extirpated in California; BBS shows *V. b. arizonae* stable in Arizona.	Somewhat oversampled in NW Mexico because of late summer singing.
*Icterus spurius*	+8.48	BBS: significant survey-wide declines; sharpest declines in southern states.	Common new breeder for Sinaloa and southern Sonora; migrates further south to molt
*Stelgidopteryx serripennis*	+8.21	BBS: significant declines in the west, but not in Canada.	Our AI ratio overestimates increases in breeders because breeders and molt migrants were not distinguished.
*Oreothlypis lucae*	+7.38	BBS: slight, non-significant increase in the US; but large declines are reported in the mid 1980s for a long-term study in AZ (Lamm, 1991).	Common new breeder for Sinaloa, but our AI ratio does not distinguish breeders and molt migrants.
*Icteria virens*	+1.87	BBS: Mostly stable in the west.	Common breeder with more available habitat.
*Geothlypis trichas*	−3.17	BBS: Stable in the west	Occasional breeder in interior wetlands; common along the coast where we seldom collected.

Yellow-billed Cuckoos have disappeared from most of their historic breeding range in the western US and Canada (Goldwasser, Gaines & Wilbur, 1980; Gaines & Laymon, 1984; Laymon & Halterman, 1987). Although much riparian habitat has been lost in the Southwest US, the loss of cuckoos from northern parts of their western breeding range is inconsistent with their decline being driven by losses of breeding habitat because landscape changes in the north have increased ecotonal habitats they prefer for breeding. Although historically widely distributed in the west, the western population of cuckoos was probably never very large because they were restricted to riparian corridors and human created ecotones. The density of cuckoos now breeding in the irrigated habitats of coastal NW Mexico easily seems high enough to account for the numbers that previously bred in the western US and Canada. Further, the decline in the much larger population of cuckoos in eastern North America (Sauer et al., 2013), suggests that loss of winter habitat may explain their continent-wide decline in breeding numbers, even though they surely have increased as breeding birds in coastal NW Mexico.

For the endangered Least’s Bell’s Vireo (*Vireo bellii pusillus*) of California the situation is less compelling, but shortstopping seems worth considering given the remarkable abundance of Bell’s Vireos that now breed in irrigated regions of NW Mexico. The California population has responded positively to habitat improvements and to cowbird control, which weakens a case for a causal link between the expanding population in NW Mexico and declines in California (Kus, 1998; Kus & Whitfield, 2005). *Pusillus* supposedly winters only in Baja California, but a small change in the fall migratory orientation of *pusillus* that breed in California would take them to the excellent new breeding habitat created by irrigation in coastal Sonora and Sinaloa.

With an average of almost $2.5 m spent on recovery efforts each year for Least Bell’s Vireos (Restani & Marzluff, 2001), it seems important to assess whether their decline in the US might be linked to their increase in NW Mexico. To assess the source of the Bell’s Vireos now breeding in Sinaloa, we attempted to assign 25 new Sineloa specimens to race: 18 better matched *V*.*b*. *arizonae*, but 7 better matched *V*.*b*. *pusillus*. Distinguishing these races is difficult, so this conclusion should be further evaluated using genetics or geolocators (Klicka, 2014). If further genetic work using SNPs (Klicka, 2014) discloses *pusillus* genotypes in the now large breeding populations in NW Mexico, then habitat restoration in California could be competing with the recently developed irrigated habitat in coastal Sonora and Sinaloa. Alternatively, if the *V. bellii* now breeding in coastal NW Mexico all carry *arizonae* SNPs, then there would be no reason to think shortstopping in Sinaloa and Sonora has contributed to the decline of *pusillus* in California.

Unlike cuckoos and vireos, breeding Cliff Swallows remain abundant in Canada and the western USA, but declines have been substantial since the Breeding Bird Survey was begun. Our AIs greatly underestimate the increase in Cliff Swallows, and the numbers we see breeding in NW Mexico easily seem sufficient to account for their decline in Canada and the western US.

Finally, Orchard Orioles were not previously thought to breed in NW Mexico (Miller et al., 1959). Not only are they common breeders in irrigated farmlands of this region (Rohwer & Wood, 2013), but their breeding densities around irrigated alfalfa fields similar to those found in the best breeding habitats in Kansas and Louisiana. The BBS shows significant survey-wide declines for Orchard Orioles, but their sharpest declines have been in southern states (Table 5). Because they are patchily distributed as breeding birds in Mexico and in the USA, we have little sense of whether the numbers now breeding in the irrigated habitats of NW Mexico are sufficient to account for the declines in breeding numbers to the north.

### Conclusions

Widespread irrigation has enormously increased the percentage of mesic land cover in coastal NW Mexico, leaving less than 40% of the land area in dry habitat. This change has increased the diversity and abundance of small birds breeding in this region, without resulting in the loss of species dependent on the original thorn forest habitat. The greatest value of discovering new breeding populations or large increases in breeding numbers of some migrants in this region is the implication that these population increases of Mexico could be linked, through winter population regulation, to declines in breeding numbers in the US and Canada. Given the sums being invested in these populations in the US (Restani & Marzluff, 2001), further exploring this possible linkage seems urgent. In general, both the connections between events affecting winter and summer ecology of migrants (Wilcove & Wikelski, 2008) and the degree to which habitat distributions may be affected through domino effects of social behavior over landscape and, possibly, range-wide scales (Graves, 1997; Rohwer, 2004; Studds, Kyser & Marra, 2008) seems underappreciated. If breeding numbers to the north are causally related to the development of new breeding populations in Mexico, then recovery investments for those species in the US and Canada should be redirected to other management priorities.

## Supplemental Information

10.7717/peerj.1187/supp-1 Supplemental Information 1 Early collection NW Mexican birds 1870–1979

10.7717/peerj.1187/supp-2 Supplemental Information 2 Recent collection NW Mexico birds 1980–2011

## Additional Information and Declarations

### Field Study Permissions

The following information was supplied relating to field study approvals (i.e., approving body and any reference numbers): Collecting for this project was conducted under scientific collection permits issued by the Secretaría de Medio Ambiente y Recursos Naturales (SEMARNAT) to the Facultad de Ciencias (B. Hernández-Baños FAUT 0169) of UNAM.
